# Supplementary material for: Accuracy of ankle-brachial index in screening for peripheral arterial disease in people with diabetes
Source: PLoS One. 2024 Oct 24;19(10):e0309083. doi: 10.1371/journal.pone.0309083 (PMC11501029; doi:10.1371/journal.pone.0309083)
Supplement: Attachment — Submitted filename: Response to Reviewers.pdf [file pone.0309083.s002.pdf]

Dear Dr. Biswabandhu Jana,

We sincerely appreciate your meticulous review of our manuscript. We would like to clarify a few points in response to your comments.

Initially, we corrected the statement that our study would be the first with this focus after thoroughly reviewing the works included in the systematic reviews available. In the latest systematic review published by Chuter et al. (2024), we observed that a recent study did not clarify whether the participants were recruited from the community, while another could not be thoroughly reviewed. Our research group did not conduct a systematic review on the subject, so we modified this sentence. The study referenced in link 10.4103/jfmpc.jfmpc_1546_20, which has similar characteristics to ours, is a cross-sectional observational study conducted during a workshop for diabetic patients in the outpatient department of a hospital, without specifying whether the participants were already being followed or were recruited from the community.

We believe that our study contributes to the existing literature by investigating the performance of the ABI in screening PAD in a community context, where the characteristics of recruited participants differ considerably from specialized centers, even among asymptomatic individuals. In study design, the recruitment location of participants is crucial, as an overly specific selection may not adequately represent the general population, compromising the applicability of the results in screening tests. It is known that in specialized centers, the prevalence of the disease may differ substantially from that found in the community population. More severely affected patients may be included in areas with higher disease prevalence, which may overestimate the test's performance.

Additionally, based on the systematic reviews published on the topic, we identified that the current evidence on the role of ABI <0.9 as a potentially helpful test for diagnosing PAD is based on a limited number of studies (n = 3), with moderate to high risks of bias. Therefore, we believe there is a demand for studies with better-defined methodologies to expand our understanding of the performance of this test, considering the significant impact of undiagnosed and inadequately managed PAD in individuals with diabetes.

Sincerely,

The Authors

Dear Reviewer #1,

We appreciate your careful review of our manuscript. We have reviewed all the texts and accepted and incorporated your correction suggestions. In the revised manuscript, these changes are appropriately highlighted.

Regarding your question about how our study population differs from populations in studies conducted in hospital settings, we would like to clarify a few points. The accuracy of a diagnostic tool depends not only on its sensitivity and specificity but also on the prevalence of the disease in the studied population. In studies conducted in specialized centers, the prevalence and severity of the disease significantly differ from the community population, which can lead to an overestimation of the test's performance.

This justification has been better elaborated and is available in the introduction of the revised work.

Sincerely,

The authors.

Dear Reviewer #2,

We appreciate your careful review of our manuscript. We have reviewed the entire article. To enhance the presentation of our results, in addition to evaluating the diagnostic accuracy of the text, we have added a Bayesian regression model to explore the diagnostic performance of ABIs in specific population subgroups. The detailed analyses are described in the Methods section, and their graphical representations have been included as new figures in the article.

Sincerely,

The authors.
